# Decoding the mitochondrial connection: development and validation of biomarkers for classifying and treating systemic lupus erythematosus through bioinformatics and machine learning

**DOI:** 10.1186/s41927-023-00369-0

**Published:** 2023-12-04

**Authors:** Haoguang Li, Lu Zhou, Wei Zhou, Xiuling Zhang, Jingjing Shang, Xueqin Feng, Le Yu, Jie Fan, Jie Ren, Rongwei Zhang, Xinwang Duan

**Affiliations:** Department of Rheumatology and Immunology, the Second Affiliated Hospital of Nanchang University, Nanchang, Jiangxi 330006 China

**Keywords:** Systemic Lupus Erythematosus (SLE), Mitochondria-related genes (MRGs), Biomarkers, Bioinformatics, Machine learning

## Abstract

**Background:**

Systemic lupus erythematosus (SLE) is a multifaceted autoimmune disease characterized by clinical and pathological diversity. Mitochondrial dysfunction has been identified as a critical pathogenetic factor in SLE. However, the specific molecular aspects and regulatory roles of this dysfunction in SLE are not fully understood. Our study aims to explore the molecular characteristics of mitochondria-related genes (MRGs) in SLE, with a focus on identifying reliable biomarkers for classification and therapeutic purposes.

**Methods:**

We sourced six SLE-related microarray datasets (GSE61635, GSE50772, GSE30153, GSE99967, GSE81622, and GSE49454) from the Gene Expression Omnibus (GEO) database. Three of these datasets (GSE61635, GSE50772, GSE30153) were integrated into a training set for differential analysis. The intersection of differentially expressed genes with MRGs yielded a set of differentially expressed MRGs (DE-MRGs). We employed machine learning algorithms—random forest (RF), support vector machine (SVM), and least absolute shrinkage and selection operator (LASSO) logistic regression—to select key hub genes. These genes’ classifying potential was validated in the training set and three other validation sets (GSE99967, GSE81622, and GSE49454). Further analyses included differential expression, co-expression, protein-protein interaction (PPI), gene set enrichment analysis (GSEA), and immune infiltration, centered on these hub genes. We also constructed TF-mRNA, miRNA-mRNA, and drug-target networks based on these hub genes using the ChEA3, miRcode, and PubChem databases.

**Results:**

Our investigation identified 761 differentially expressed genes (DEGs), mainly related to viral infection, inflammatory, and immune-related signaling pathways. The interaction between these DEGs and MRGs led to the identification of 27 distinct DE-MRGs. Key among these were FAM210B, MSRB2, LYRM7, IFI27, and SCO2, designated as hub genes through machine learning analysis. Their significant role in SLE classification was confirmed in both the training and validation sets. Additional analyses included differential expression, co-expression, PPI, GSEA, immune infiltration, and the construction of TF-mRNA, miRNA-mRNA, and drug-target networks.

**Conclusions:**

This research represents a novel exploration into the MRGs of SLE, identifying FAM210B, MSRB2, LYRM7, IFI27, and SCO2 as significant candidates for classifying and therapeutic targeting.

**Supplementary Information:**

The online version contains supplementary material available at 10.1186/s41927-023-00369-0.

## Background

Systemic lupus erythematosus (SLE) is a chronic inflammatory autoimmune disease that primarily affects females of childbearing age, with a male to female ratio of 1:9, and is characterized by the production of a variety of autoantibodies that can affect multiple organs [1, 2]. The prevalence of SLE in adults globally ranges from 30 to 150 per 100,000 individuals, with incidence ranging from 2.2 to 23.1 per 100,000 individuals per year, and varies by region and ethnicity [3]. Despite improvements in SLE survival rates over the past few decades, premature mortality remains two to three times higher than that of the general population [4], highlighting the persistent challenges in managing this disease.

Firstly, the clinical presentation of SLE is highly heterogeneous, with an increasing number of atypical cases, leading to confusion in classification and delays in treatment initiation. Secondly, treatment of SLE typically involves immunosuppressive therapy, such as broad-spectrum immunosuppressants and glucocorticoids. However, these treatments are not uniformly effective, and several patients may experience relapse. Additionally, long-term use of these drugs can cause organ damage and have substantial toxic effects. SLE results from different pathogenic mechanisms, leading to a wide range of clinical manifestations and cellular and molecular foundations. Therefore, uncovering the underlying molecular mechanisms of SLE is of great clinical significance for its classification and management.

Recent research has shown that mitochondrial dysfunction is a critical factor in the pathogenesis of SLE [5–7]. Mitochondria, which are autonomous double-membrane organelles, play a key role in many intracellular processes such as oxidative phosphorylation, amino acid biogenesis, fatty acid catabolism, calcium homeostasis, and apoptosis [8]. Therefore, mitochondria are crucial for maintaining normal cellular physiology. Structural damage and functional alterations of mitochondria can cause various pathological states, including damage to mitochondrial DNA (mtDNA), changes in mitochondrial dynamics, abnormal mitochondrial biogenesis and energy metabolism, oxidative stress, and inflammatory reactions. All of these factors contribute to the pathogenesis of SLE. In addition, mitochondria are involved in the cell death pathway, including apoptosis, necrosis, and NETosis in neutrophil granulocytes, which is another mechanism that leads to the emergence of SLE. Other pathways, such as changes in mitochondrial dynamics and mitophagy, also contribute to the onset of SLE. Targeting cellular metabolic changes in mitochondria has been shown to have therapeutic effects [9, 10]. Hence, the genes associated with mitochondrial function, or mitochondrial-related genes (MRGs), may hold vital clues for the molecular mechanisms of this disease.

Moreover, with recent advances in bioinformatics and machine learning, we now have the capability to delve deeper into a wide range of molecular mechanisms involved in SLE, analyzing high-throughput genetic data to elucidate disease progression and potentially identify novel biomarkers for classification and treatment [11–14]. Despite these capabilities, however, the genetic investigation of MRGs in the context of SLE remains largely unexplored, and there are currently no established models that evaluate mitochondrial function in the disease. Therefore, this study was undertaken with the objective to delve into the molecular characteristics of MRGs in SLE through bioinformatics analysis and machine learning techniques. Our intent was not only to discover new biomarkers for the development and validation of a classification model but also to identify potential therapeutic targets for SLE.

## Methods

### Data acquisition and processing

The GEOquery R package [15] was employed to download microarray datasets relevant to SLE from the Gene Expression Omnibus (GEO) online database, a public functional genomics database that archives and freely disseminates microarray and other forms of high-throughput data [16]. The following filtering criteria were used: (1) The test specimens should be from humans; (2) The tissue used for sequencing should be peripheral blood mononuclear cell (PBMC); (3) The independent expression profiles of training set should be from the same sequencing platform to facilitate integration. Based on the above criteria, seven datasets (GSE61635, GSE50772, GSE30153, GSE99967, GSE81622, and GSE49454) relevant to SLE were finally included in this study. The GSE61635 dataset (GPL570 platform) included 79 SLE and 30 normal PBMC samples, the GSE50772 dataset (GPL570 platform) included 81 SLE PBMC samples, and the GSE30153 dataset (GPL570 platform), which included 17 SLE and 9 normal PBMC samples, were merged and used as a training set for subsequent analyses. The batch effects of the dataset were also removed using the R function ComBat, which is part of the sva package [17]. The GSE99967 dataset (GPL21970) included 42 SLE and 17 normal PBMC samples, the GSE81622 dataset (GPL10558) contained 30 SLE and 25 normal PBMC samples, and the GSE49454 dataset (GPL10558) comprised 157 SLE and 20 normal PBMC samples were selected for validation analysis. All raw data in our investigation was subjected to normalization and adjustment for background, and we also cross-referenced all probe names with their respective gene symbols.

Additionally, the 1136 mitochondria-related genes (MRGs) included in Supplementary Data S1 were selected from the human MitoCarta 3.0 database (https://www.broadinstitute.org/mitocarta/mitocarta30-inventory-mammalian-mitochondrial-proteins-and-pathways) [18].

### Identification of differentially expressed genes (DEGs)

The limma package in R program [19] was used to evaluate differentially expressed genes (DEGs) between SLE and healthy controls, using cutoffs for adjustment: p-value < 0.05 and FC (fold changes) > 1.5. The volcano plot and heatmap of the DEGs were visualized using the ggplot2 R package [20].

### Functional enrichment analysis of DEGs

To gain a better understanding of the role of DEGs in SLE, we conducted Gene Ontology (GO) and Kyoto Encyclopedia of Genes and Genomes (KEGG) pathway analyses using clusterProfiler, an R-package [21]. For data that has been adjusted for the false discovery rate (P < 0.05), the Benjamini-Hochberg multiple correction method was utilized. The GOplot R program [22] was used to plot the top 10 findings from GO and KEGG.

### Identification of mitochondria-related hub genes based on machine learning algorithm

Interaction of DEGs with MRGs yielded differentially expressed MRGs (DE-MRGs), which were then used to screen mitochondria-related hub genes. To filter out potential hub genes from DE-MRGs, we employed three machine learning techniques, namely least absolute shrinkage and selection operator (LASSO), support vector machine (SVM), and random forest (RF), each with 10-fold cross-validation for model stability, using the R packages glmnet [23], e1071 [24] and caret [25], and randomForest [26]. The overlapping genes obtained from the three algorithms were considered as the final set of hub genes.

### Construction and validation of the classification model for SLE

We utilized the neuralnet R package [27] to create an artificial neural network (ANN) model to differentiate individuals with SLE from healthy controls based on the expression patterns of specific mitochondrial-related genes. To evaluate the classification accuracy of the model, we calculated receiver operating characteristic (ROC) curves and area under the curve (AUC) in both the training and validation sets, generated with the pROC R package [28]. Moreover, the decision curve analysis (DCA) and calibration curve were applied to evaluate the accuracy and practical applicability of the classification model with the rms R package (https://CRAN.R-project.org/package=rms). The purpose of this process was to explore the potential roles these genes could play in SLE pathogenesis, operating on the premise that genes demonstrating high classification efficacy might serve as promising biomarkers and have significant implications for understanding the disease mechanism.

### Differential expression and co-expression analyses of hub genes

We conducted differential expression and co-expression analyses on the expression data of hub genes from the training set using the R packages igraph [29] and reshape2 [30]. These analyses allowed us to investigate the expression profiles of hub genes. Additionally, we used the RCircos package [31] to visualize the chromosomal locations of the hub genes.

### Protein-protein interaction (PPI) analysis of hub genes

In order to further reveal the potential relationships between proteins encoded by these hub genes, we performed a PPI enrichment analysis for core shared genes using GeneMANIA [32]. A combined score > 0.4 interaction was considered statistically significant. GeneMANIA is an online tool that generates hypotheses about gene functions, evaluates gene lists, and ranks genes by priority for functional testing.

### Immune Infiltration analysis of Hub genes

The relationship between immune infiltration and the development and progression of SLE is well established. Thus, we evaluated the relationship between the hub genes and the immune cells using the CIBERSORT algorithm (https://cibersort.stanford.edu/). CIBERSORT is a deconvolution algorithm that calculates the proportion of different immune cell types based on the expression levels of immune cellrelated genes. The output results of 22 infiltrating immune cells were integrated, and nonparametric correlations (Spearman) method was used to determine the correlation between the hub genes and immune-infiltrated cells, which was visualized using the R packages ggplot2 [20].

### Gene set enrichment analysis (GSEA) of hub genes

In addition, we conducted GSEA analysis using the clusterProfiler program [21] to identify the biological process involving the hub genes. GSEA is a method based on functional categories that could calculate the enrichment score of gene sets and discover different functional phenotypes. Based on the median expression of the hub genes, SLE patients were categorized into high- and low-expression groups. Then, we used GSEA to compare the biological pathways between the two groups. The “h.all.v2022.1.Hs.entrez.gmt” file was downloaded for GSVA analysis from the Molecular Signatures Database (MSigDB, http://software.broadinstitute.org/gsea/msigdb). Enriched gene sets with nominal P values of < 0.05, |normalized enrichment scores (NES) | > 1 and FDR q values of < 0.25 were considered significant.

### Regulation networks of hub genes construction

We used the ChIP-X Enrichment Analysis 3 (ChEA3) platform (https://maayanlab.cloud/chea3/) to submit the hub genes for transcription factors (TFs) prediction [33]. ChEA3 is a TF prediction database, which integrates ENCODE, ReMap, GTEx, Enrichr, ARCHS4 and Literature ChIP databases. We also used the miRcode database (http://www.mircode.org) [34] to predict the hub genes-targeted miRNAs. Cytoscape [35] was used to construct and visualize the TF-mRNA and miRNA-mRNA networks. Additionally, we used the R package networkD3 [36] to display a Sankey diagram depicting the anticipated biological activities of small molecules associated with the hub genes from the PubChem database (https://pubchem.ncbi.nlm.nih.gov/) [37].

### Statistical analysis

Unless specified otherwise, all analyses and visualizations were conducted using R software (version.4.2.1). The Student’s t-test was utilized for the evaluation of variables adhering to a normal distribution, whereas the Wilcoxon rank-sum test was employed for variables that were not normally distributed. Normality was assessed using the Shapiro-Wilk test. A two-sided P value of less than 0.05 was considered statistically significant. Concurrently, for the identification of differentially expressed genes, criteria for statistical significance were set as an adjusted P value (adj.P) less than 0.05 and a fold change (FC) greater than 1.5.

## Results

### Identification of DEGs in SLE

**Fig. 1:**
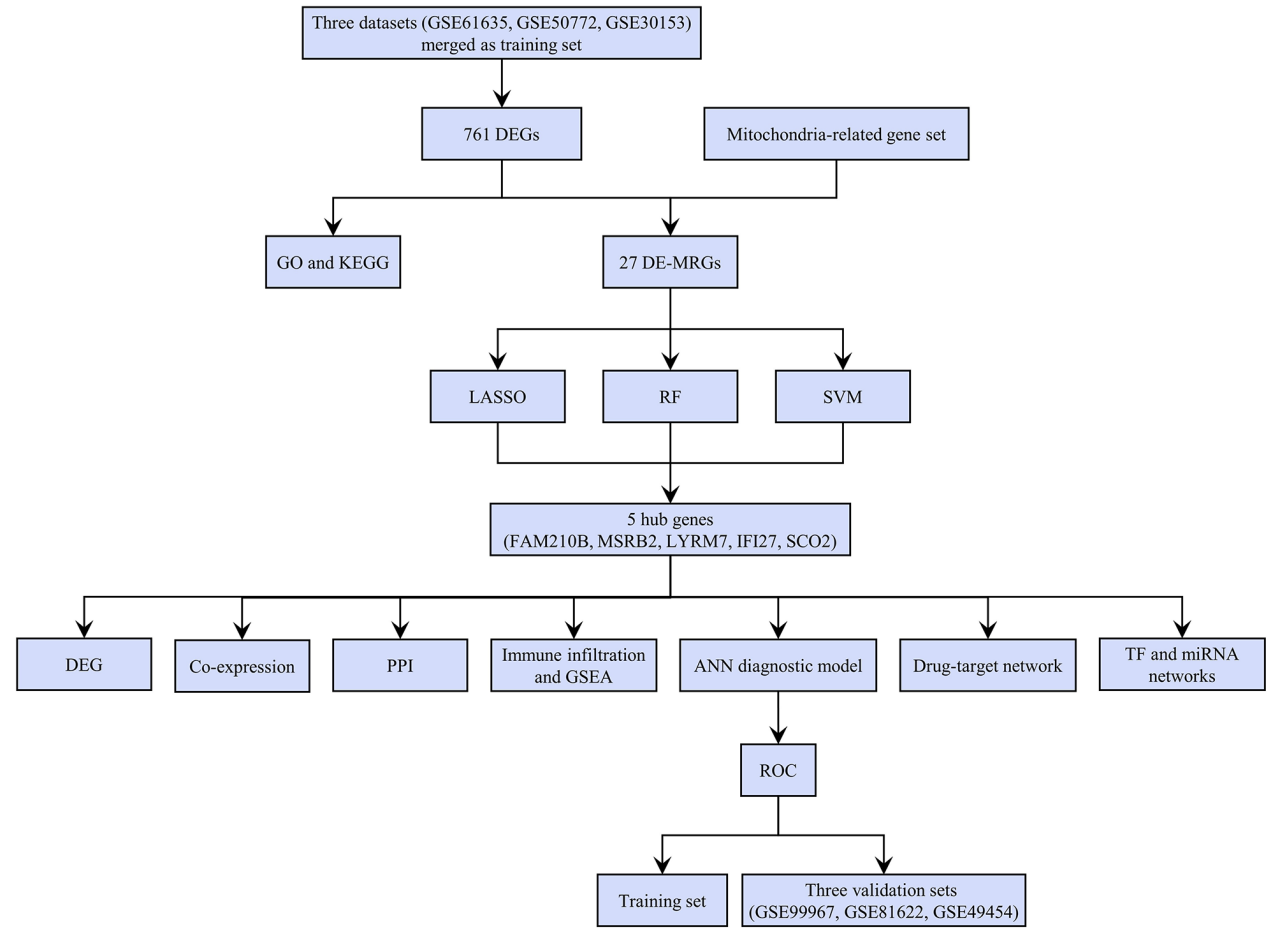
The study flow chart

**Fig. 2:**
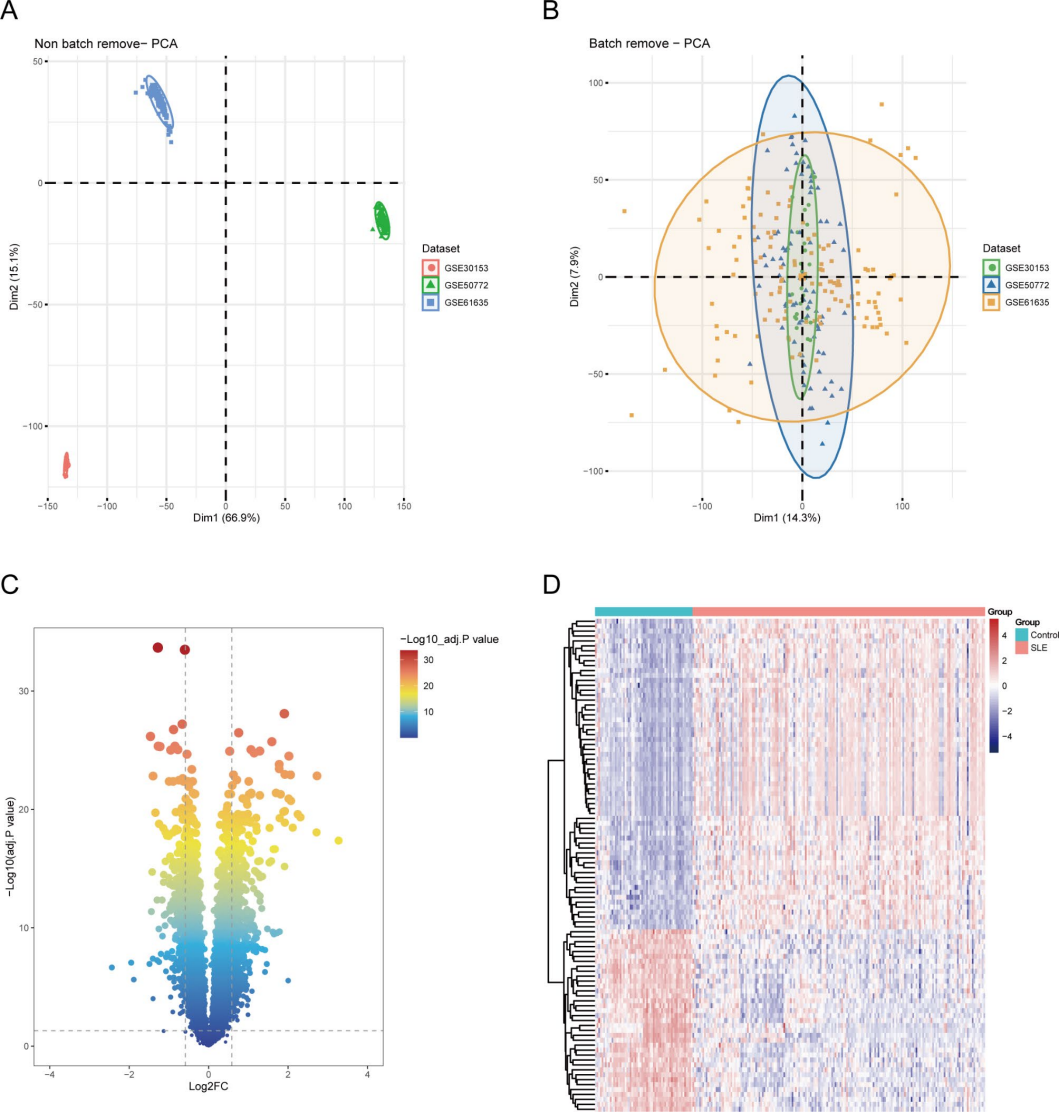
Identification of DEGs. **A**. PCA results for the combined expression profile before and after Combat. **B**. Volcano plot displaying DEGs, with fold change > 1.5 and adjusted p-value < 0.05. **C**. Heatmap of the top 100 DEGs ranked by adjusted *p*-values, indicating gene expression trends in different PBMC. DEGs: differentially expressed genes; PCA: principal component analysis; PBMC: peripheral blood mononuclear cell

Three microarray datasets (GSE61635, GSE50772, GSE30153) comprising of 177 SLE and 39 normal PBMC samples were merged and utilized as the training set for this study. Figure 1 depicts a comprehensive flowchart of the research procedure. Principal component analysis (PCA) was employed to identify inherent batch differences in the training set (Fig. 2A). To improve the efficacy of the subsequent analysis, we used the “ComBat” algorithm to address the batch effects. The batch-correction methods successfully eliminated the batch effects to a certain degree (Fig. 2B). Then, differential expression analysis was performed on the training set to screen for differentially expressed genes (DEGs). A total of 761 significant DEGs associated with SLE were identified, including 446 upregulated and 315 downregulated genes, based on significance criteria (Supplementary Data S2). The results were shown in a volcano plot (Fig. 2C). In addition, the top 100 DEGs, ranked according to adjusted P-values, were displayed in a heatmap (Fig. 2D).

### Functional annotation and pathway enrichment of DEGs

**Fig. 3:**
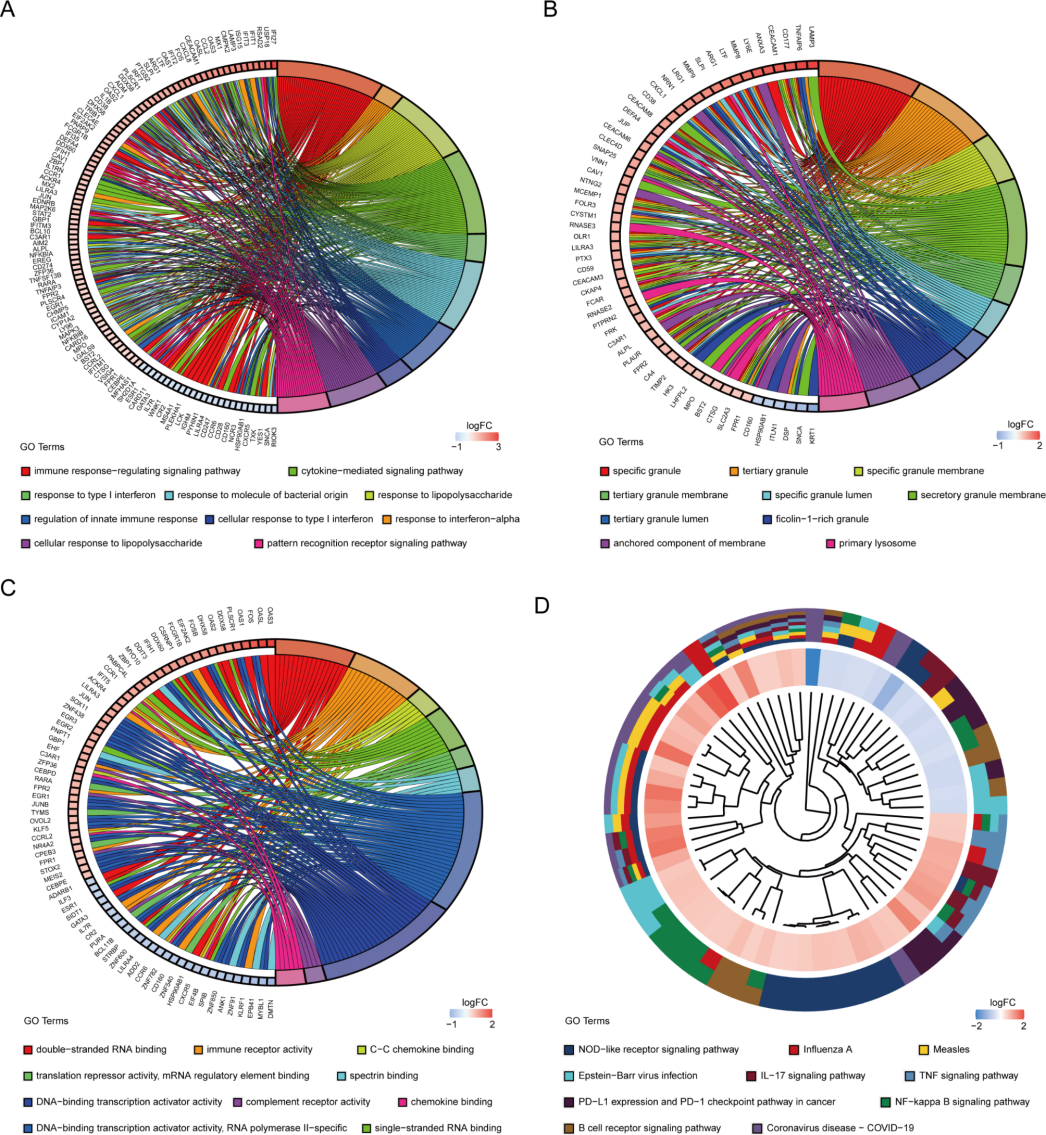
Functional annotation and pathway enrichment of DEGs. **A**. Top 10 biological process (BP) GO pathways. **B**. Top 10 cellular component (CC) GO pathways. **C**. Top 10 molecular function (MF) GO pathways. **D**. Top 10 KEGG pathways. DEGs: differentially expressed genes; GO: gene ontology; KEGG: Kyoto Encyclopedia of Genes and Genomes

According to GO-BP analysis, the DEGs were considerably enriched in activities associated with immune responses, such as response to interferon-alpha, response to lipopolysaccharide, response to type I interferon, response to molecule of bacterial origin, cellular response to type I interferon, cellular response to lipopolysaccharide, immune response-regulating signaling pathway, regulation of innate immune response, and pattern recognition receptor signaling pathway (Fig. 3A). The findings of the GO-CC analysis were primarily granules and lumens of various immune cells (Fig. 3B). The double-stranded RNA binding, chemokine binding, single-stranded RNA binding, spectrin binding, immune receptor activity, translation repressor activity, DNA-binding transcription activator activity, and complement receptor activity were the key outcomes of enrichment analysis in GO-MF (Fig. 3C). Besides, investigation of the KEGG pathway analysis primarily suggested that these DRGs were involved in virus-related diseases, including Influenza A, Measles, Epstein-Barr virus infection, and Coronavirus disease - COVID-19, and inflammatory and immune-related signaling pathways such as NOD-like receptor signaling pathway, NF-kB signaling pathway, IL-17 signaling pathway, TNF signaling pathway, PD-L1 expression, and PD-1 checkpoint pathway, and B cell receptor signaling pathway (Fig. 3D).

### Identification of hub genes for SLE

**Fig. 4:**
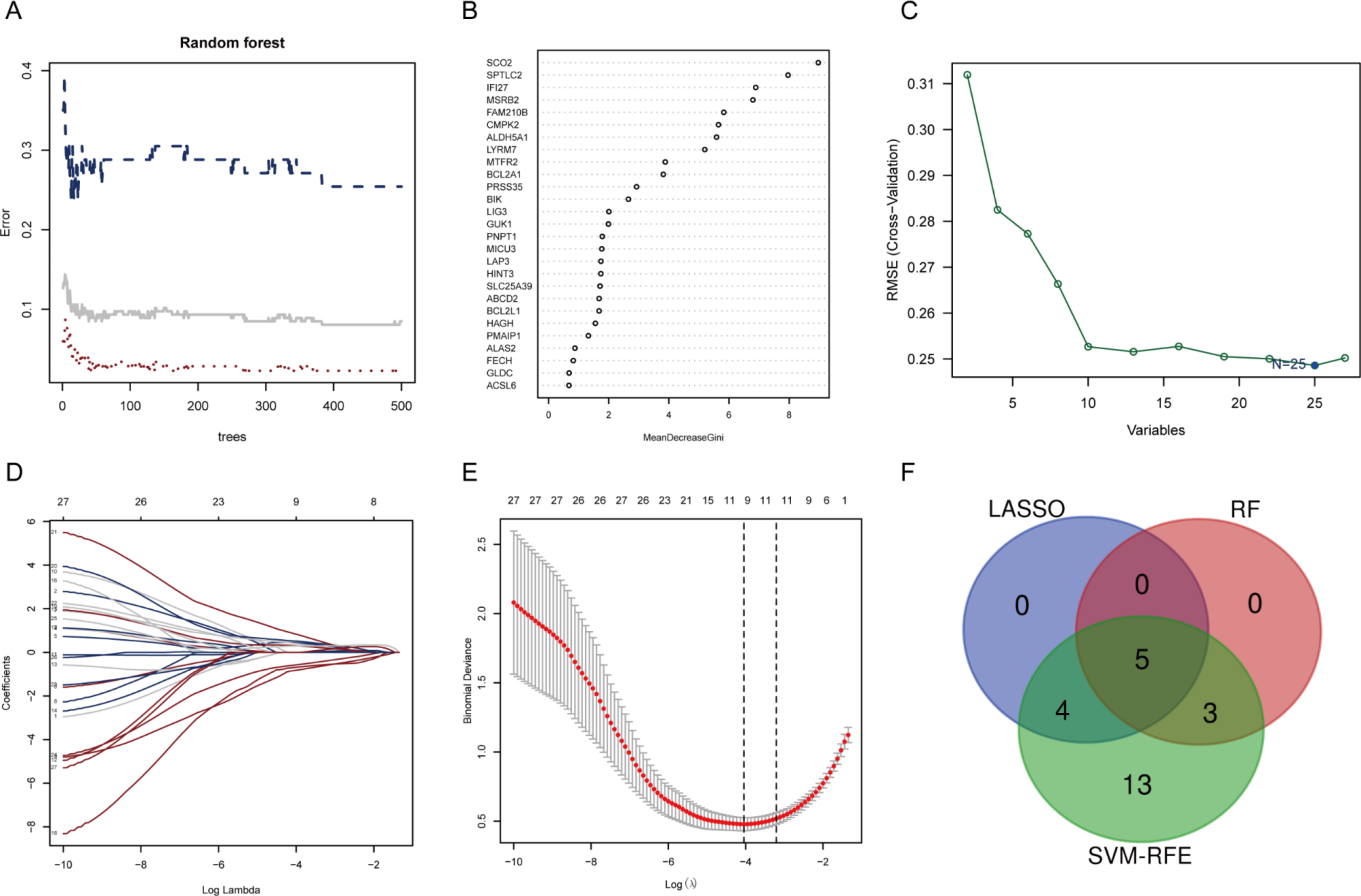
Identification of hub genes for SLE. **A-B**. RF to screen key characteristic genes. **(A)** The influence of decision tree number on error rate. **(B)** Gini importance measure of characteristic MRGs. **C-E**. LASSO logistic regression to screen key characteristic genes. **C**. RMSE of key characteristic gene combination. D. LASSO coefficient spectrum of nine genes. **E**. Optimal penalization coefficient lambda selection. **F**. Venn diagram of key characteristic genes; overlapping genes selected as hub genes. SLE: systemic lupus erythematosus; RF: random forest; MRGs: mitochondria-related genes; RMSE: root-mean-square error; LASSO: least absolute shrinkage and selection operator

Following the analysis of the interaction between DEGs and MRGs, a total of 27 DE-MRGs were identified and listed in Supplementary Data S3. Gene expression levels of these DE-MRGs were then employed as characteristics by the three machine learning methods to distinguish between SLE and healthy controls. The lowest error rate and most stable results were achieved using 383 trees in the RF classifier (Fig. 4A). Therefore, to estimate the dimensional significance of the 27 DE-MRGs, we settled on 383 trees as the last parameter in the RF classifier, and the resulting MeanDecreaseGini findings are shown in Fig. 4B. 8 candidate hub genes were discovered, including SCO2, SPTLC2, IFI27, MSRB2, FAM210B, CMPK2, ALDH5A1, and LYRM7 from the DE-MRGs after settling on a screening threshold of 5 based on the significance of the MeanDecreaseGini result. Besides, as shown in Fig. 4C, the SVM model has the minimum classification error [minimal root-mean-square error (RMSE) = 0.248] in the condition of 25 candidate genes, so 25 genes were recognized as key characteristic genes from the DE-MRGs, including IFI27, SPTLC2, SCO2, MSRB2, CMPK2, LYRM7, ALDH5A1, FAM210B, BCL2A1, PRSS35, MTFR2, BIK, SLC25A39, LAP3, HINT3, ABCD2, LIG3, GUK1, MICU3, HAGH, PNPT1, BCL2L1, ACSL6, PMAIP1, and ALAS2. In LASSO logistic regression, the optimal lambda of LASSO logistic regression was 0.0175054 (Fig. 4D and E), thus extracting nine key characteristic genes from the DE-MRGs, comprised of BCL2A1, BIK, FAM210B, IFI27, LYRM7, MSRB2, MTFR2, PRSS35, and SCO2. Finally, FAM210B, MSRB2, LYRM7, IFI27, and SCO2, overlapping genes by the three algorithms, were selected as hub genes (Fig. 4F).

### Performance of hub genes to classify SLE

**Fig. 5:**
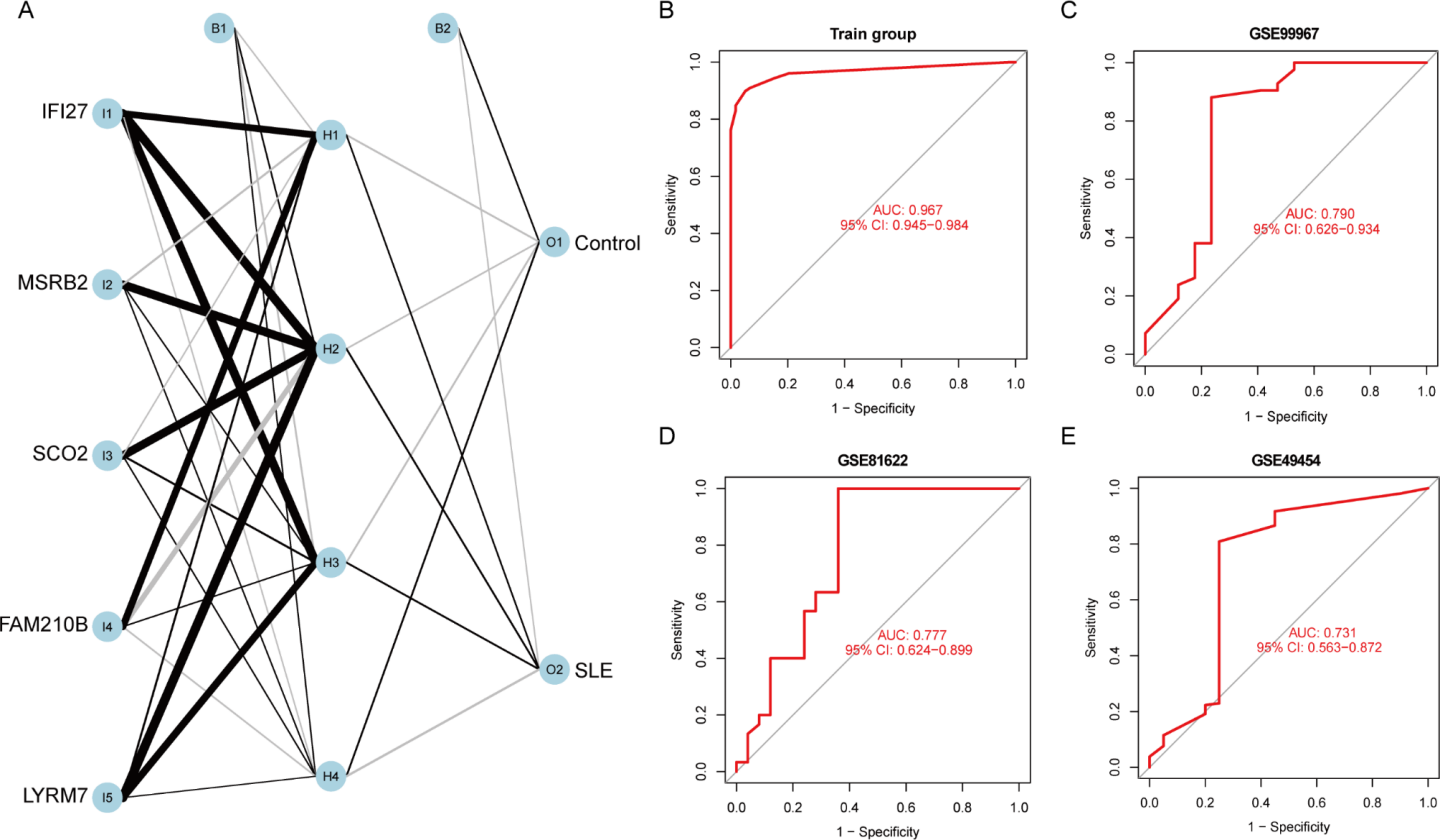
Performance of hub genes to classify SLE. **A**. Disease classification model constructed by an ANN. **B-E**. ROC curves for model classification of SLE in training and three validation sets. SLE: systemic lupus erythematosus; ANN: artificial neural network; ROC: receiver operating characteristic

Next, an ANN model for classifying SLE was developed utilizing five hub genes, achieving an AUC of 0.967 (95% CI 0.945–0.984) on the training set, as shown in Fig. 5A and B. Furthermore, the performance of this ANN model was tested on three distinct validation datasets (GSE99967, GSE81622, and GSE49454), and it was shown to perform well in the validation sets, with AUCs of 0.790 (95% CI 0.626–0.934), 0.777 (95% CI 0.624–0.899), and 0.731 (95% CI 0.563–0.872), respectively (Fig. 5C, D, and E); its performance was further substantiated by the calibration curve and DCA (Figure S1).

### Gene expression patterns and PPI network

**Fig. 6:**
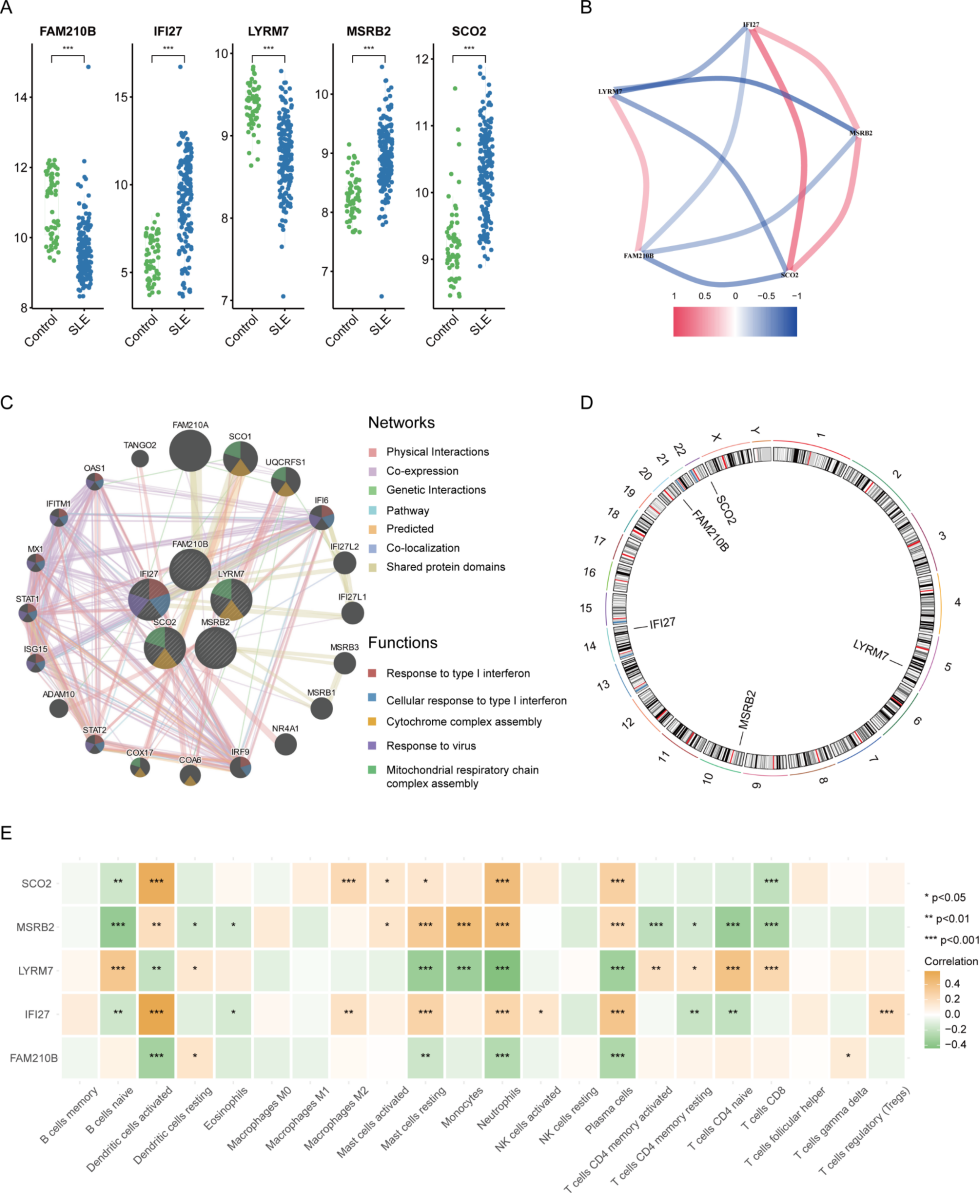
Hub genes in SLE. **A**. Differential expression levels of hub genes between SLE and healthy controls in the training set. **B**. Gene relationship network of hub genes. **C**. PPI network of hub genes and interacting proteins. **D**. Chromosomal locations of hub genes. **E**. Correlation matrix between hub genes and immune cells. SLE: systemic lupus erythematosus; PPI: protein-protein interaction

Afterwards, we examined the gene expression patterns in the training data. We found that the expression levels of IFI27, MSRB2, and SCO2 were significantly higher in SLE patients compared to normal controls, while the levels of FAM210B and LYRM7 were significantly lower, as determined by the Wilcoxon rank-sum test (Fig. 6A). The gene relationship network indicated a close link between these hub genes and suggested that IFI27, MSRB2, and SCO2 might have opposing regulatory effects compared to FAM210B and LYRM7 in the pathogenesis of the disease (Fig. 6B).

Furthermore, we constructed a PPI network for the five hub genes, which included 20 other genes such as FAM210A, SCO1, UQCRFS1, IFI6, IFI27L2, IFI27L1, MSRB3, MSRB1, NR4A1, IRF9, COA6, COX17, STAT2, ADAM10, ISG15, STAT1, MX1, IFITM1, OAS1, and TANGO2, which were mainly involved in the functions of Response to type I interferon, Cellular response to type I interferon, Cytochrome complex assembly, Response to virus, and Mitochondrial respiratory chaincomplex assembly (Fig. 6C).

Additionally, we illustrated the chromosomal locations of the five hub genes: FAM210B (chr20), MSRB2 (chr10), LYRM7 (chr5), IFI27 (chr14), and SCO2 (chr22) (Fig. 6D).

### Immune infiltration and GSEA of hub gene

**Fig. 7:**
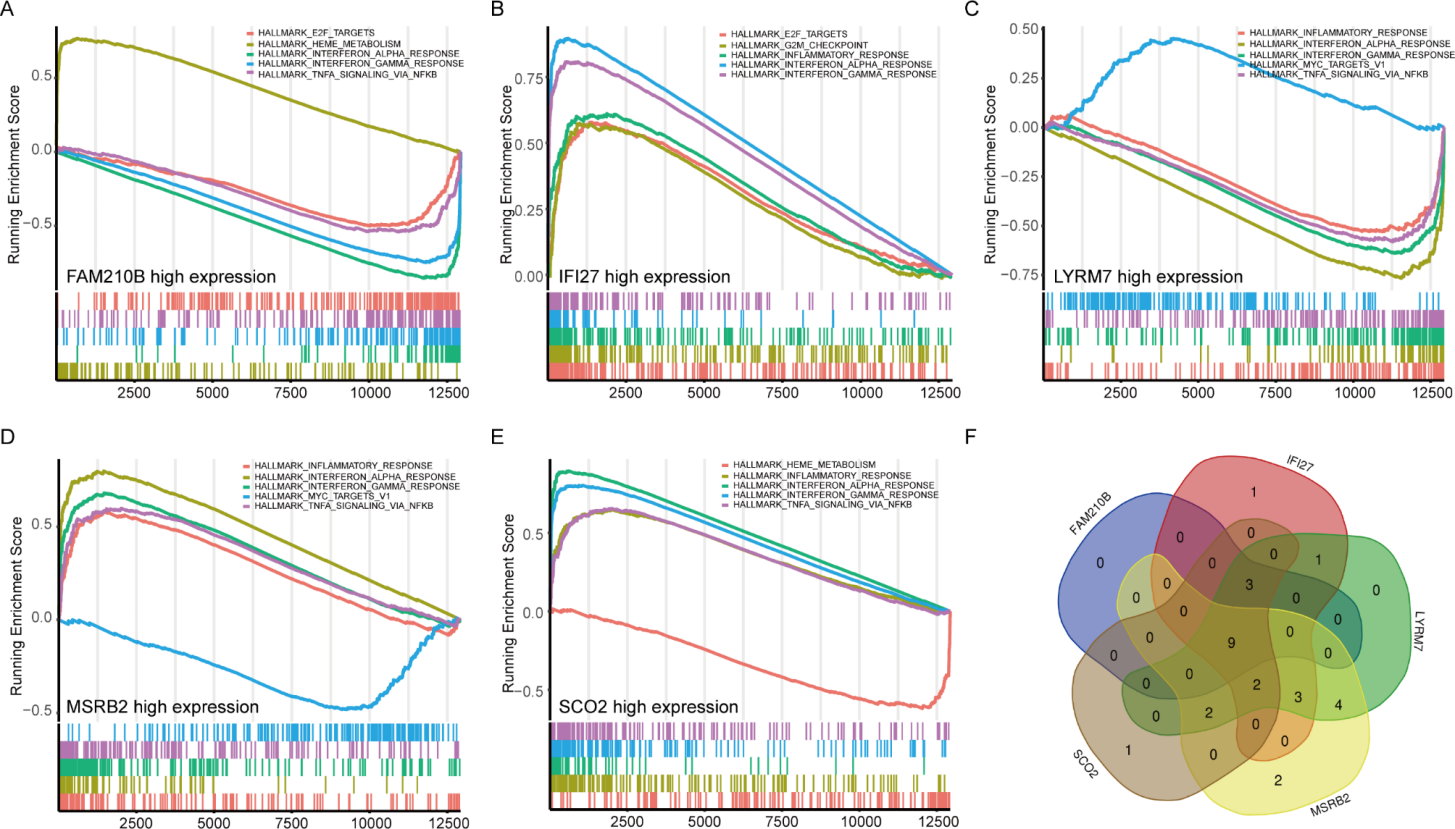
GSEA of hub genes. **A-E**. Pathways enriched in FAM210B, IFI27, LYRM, MSRB2, and SCO2. **F**. Venn diagram of shared pathways. GSEA: gene set enrichment analysis

Subsequently, we investigated the relationship between the hub genes and immune cells and noted that the core genes were closely linked with most immune cells (Fig. 6E). Furthermore, we performed GSEA of the hub genes on SLE samples and found that these genes were mainly enriched in inflammatory and immune-related signaling pathways (Fig. 7A-E). Combining the GSEA data from the hub genes revealed nine common pathways, including IL6_JAK_STAT3_SIGNALING, INTERFERON_ALPHA_RESPONSE, INFLAMMATORY_RESPONSE, COMPLEMENT, TNFA_SIGNALING_VIA_NFKB, HEME_METABOLISM, APOPTOSIS, CHOLESTEROL_HOMEOSTASIS, and INTERFERON_GAMMA_RESPONSE (Fig. 7F).

### TF-mRNA, miRNA-mRNA, and drug-target networks

**Fig. 8:**
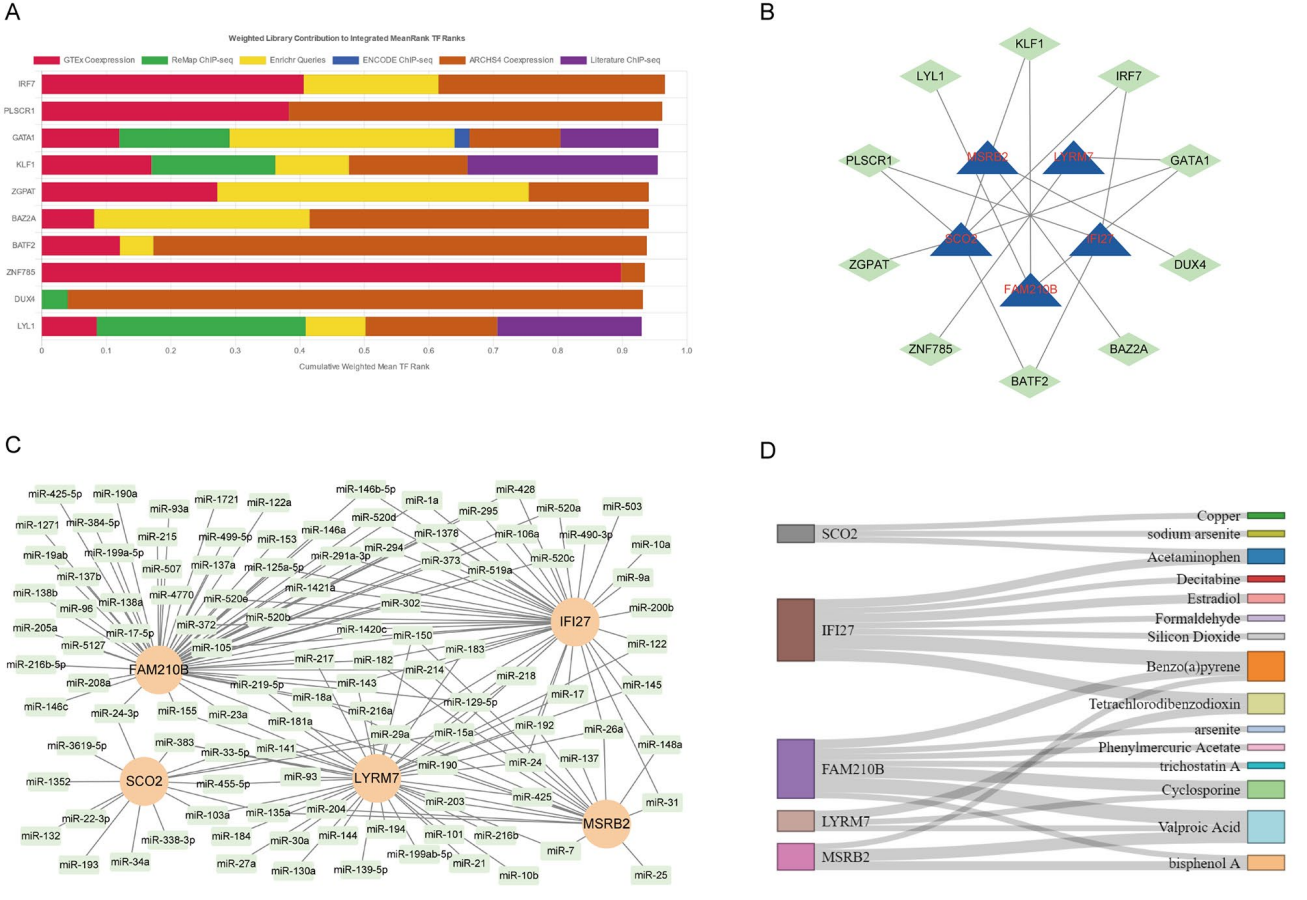
Regulation Networks of Hub Genes. **A**. TF prediction based on ChEA3 platform **B**. TF-mRNA network. **C**. miRNA-mRNA network. **D**. Drug-target network. TF: transcription factor; ChEA3: ChIP-X Enrichment Analysis 3

Additionally, we employed the ChEA3 platform to determine the most important transcription factor of the hub genes (Fig. 8A). As a result, there were 316 intersecting transcription factors enriched (Supplementary Data S4), of which the top 10 TFs ranked according to mean scores were shown in Fig. 8B. Similarly, 110 miRNAs were obtained from the online database miRcode based on the hub genes (Supplementary Data S5), and the miRNA-mRNA network was constructed as shown in Fig. 8C. Furthermore, we also mined the PubChem database for hub genes associated with mitochondrial dysfunction to identify potential drug targets for treating SLE. Total drug target prediction yielded 131 hits, 55 for FAM210B, 42 for MSRB2, 23 for LYRM7, 79 for IFI27, and 25 for SCO2 (Supplementary Data S6). Copper, sodium arsenite, acetaminophen, decitabine, estradiol, formaldehyde, silicon dioxide, benzo(a)pyrene, tetrachlorodibenzodioxin, arsenite, phenylmercuric acetate, trichostatin A, cyclosporine, valproic acid, and bisphenol A, were among the 15 target drugs with evidence greater than two independent research articles indicating their interaction with the target genes (Fig. 8D).

## Discussion

Systemic lupus erythematosus (SLE) is a complex autoimmune disease that affects nearly five million individuals worldwide. Elucidating molecular pathways is crucial for the personalized classification and treatment of SLE, which is caused by a complex network of immune-inflammatory pathogenic mechanisms and many diverse variables. The mitochondrial dysfunction has been found to be strongly implicated in pathogenesis of SLE, therefore we employed bioinformatics methods to analyze MRGs and their potential functions in SLE in this study, which may contribute to a better understanding of the disease and aid in the development of reliable biomarkers for the personalized classification and effective treatment of the disease.

In the present study, three datasets containing 177 SLE and 39 normal controls were merged and analyzed as a training set. A total of 761 DEGs associated with SLE were identified, and then annotated using the research results on function-related enrichment. The findings of the GO analysis indicate that the DEGs identified in this study are primarily involved in immune-related activities, particularly in response to interferons, lipopolysaccharides, and molecules of bacterial origin. These findings are consistent with previous studies on SLE, which have demonstrated the crucial role of the immune system in the development and progression of the disease [38]. The identified DEGs may be key players in the pathogenesis of SLE, particularly in the aberrant activation of the immune system against self-antigens [39]. Besides, the KEGG pathway analysis identified virus-related pathways, including Influenza A, Measles, Epstein-Barr virus infection, and COVID-19, as well as inflammatory and immune-related signaling pathways, such as the NOD-like receptor signaling pathway, NF-kB signaling pathway, IL-17 signaling pathway, TNF signaling pathway, PD-L1 expression, and PD-1 checkpoint pathway, and B cell receptor signaling pathway. These pathways have previously been reported to be associated with the pathogenesis of SLE, and their enrichment in this study further supports their potential involvement in the development and progression of the disease [40–42]. The identified virus-related pathways also suggest that viral infections may play a role in triggering the development of SLE [43, 44]. What’s more, the GSEA results further support the findings of the KEGG pathway analysis, highlighting the enrichment of inflammatory and immune-related signaling pathways, such as complement, IL-6, JAK-STAT3, IFN-α, INF-γ, TNF-α, and NF-kB signaling pathways. These pathways have been implicated in the pathogenesis of autoimmune diseases, including SLE [45–47], and their enrichment in this study suggests that the identified DEGs may play a crucial role in the development and progression of the disease. Overall, the results of this study provide insights into the potential involvement of the identified DEGs in the pathogenesis of SLE.

Subsequently, a total of 27 DE-MRGs were identified after the intersection with MRGs and further used to screen the hub genes. In recent years, more and more studies have used machine learning related methods for hub gene selection [48]. Hence, we integrated the predictive performance of three selected machine learning classifiers (RF, SVM, and LASSO) and identify five hub genes (FAM210B, MSRB2, LYRM7, IFI27, and SCO2), which were then used to develop an ANN model for SLE classification. The model showed excellent predictive performance in the training and three validation sets, suggesting that these genes may be important biomarkers for SLE classification.

IFI27, in particular, has been associated with type I interferon-induced apoptosis [49], and may have potential as a classification marker or immunotherapeutic target for SLE [50–52]. FAM210B and LYRM7 have important roles in regulating mitochondrial energy metabolism and the stability of mitochondrial accessory factors, respectively [53, 54]. MSRB2 has been shown to play a key role in mitophagy, which is a process that scavenges ROS and promotes cell survival [55]. SCO2 is critical for the synthesis and assembly of subunits required for the functioning of respiratory complex IV, and has been implicated in both promoting ROS generation and oxidative DNA damage, as well as activating the apoptotic pathway in response to stress [56–58]. These results indicate that the identified hub genes may play important roles in the pathogenesis of SLE by contributing to mitochondrial dysfunction, oxidative stress, and cell death pathways. However, the specific roles of the hub genes in the development and progression of SLE are not yet fully understood, and further research using functional studies is necessary to elucidate the mechanisms by which these genes contribute to SLE pathogenesis.

In addition, the results of the differential expression and co-expression analyses indicated that IFI27, MSRB2, and SCO2 may have counteractive regulatory effects when compared to FAM210B and LYRM7 in the progression of SLE, which was corroborated by the outcomes of immune infiltration and GSEA. Given the strong correlation among the hub genes, we explored their shared pathways, and integration of GSEA data from the five hub genes revealed numerous inflammatory and immune-related signaling pathways closely related to SLE including IL6_JAK_STAT3_SIGNALING, INTERFERON_ALPHA_RESPONSE, and TNFA_SIGNALING_VIA_NFKB [45–47]. These findings suggest that the hub genes were involved in the pathogenesis of SLE by regulating these pathways. Moreover, the regulatory protein network linked with the hub genes also indicated their involvement in interferon signaling pathways. Thus, the identified hub genes exhibit the potential to serve as valuable biomarkers for SLE, and our findings offer important insights into the molecular mechanisms that underlie SLE, which can guide future research in this field.

In this study, we constructed regulatory networks for hub genes using transcription factors and miRNAs, providing a theoretical basis for further investigation. Besides, researchers have focused on developing targeted therapies based on these key hub genes that play a significant role in SLE pathology. Using the PubChem database, we identified prospective targeted drugs based on the mitochondria-related hub genes that could serve as a valuable reference for developing novel therapeutic strategies for SLE. Acetaminophen and cyclosporine are known to modulate mitochondrial functions and have established roles in clinical management of SLE due to their effects on mitochondrial biogenesis and autophagy, processes that are often dysregulated in SLE patients. Epigenetic mechanisms, increasingly recognized as pivotal in lupus pathogenesis, offer another therapeutic avenue. Agents like Trichostatin A, a histone deacetylase inhibitor, have been shown to alter histone acetylation, thereby potentially correcting aberrant gene expression profiles in SLE [59–62]. Decitabine, a DNA demethylating drug, may ameliorate disease symptoms by reversing pathogenic DNA methylation patterns [63]. Moreover, our analysis suggests that TCDD, despite its notoriety as a carcinogen, exhibits properties that can attenuate inflammation in SLE, which includes inhibition of immune cell proliferation and cytokine production, as well as promotion of immunosuppressive cell differentiation [64–68]. Similarly, benzo(a)pyrene’s influence on gene expression modulation points to its potential utility in reducing inflammation and pain, a therapeutic concept extrapolated from its use in other autoimmune conditions like rheumatoid arthritis [69]. Overall, these findings provide valuable insights into potential treatment options for SLE and lay a foundation for further research into the mechanisms underlying the disease.

Our research, while providing valuable insights, also inevitably has some limitations. Firstly, our analysis focused on the gene expression profiles of PBMCs and whole blood, which, though informative, may not fully encapsulate the complex pathogenesis of SLE. Investigating cell-specific expression within distinct immune cell types and other affected tissues could yield more comprehensive insights into the disease mechanisms. Secondly, the datasets we utilized were generated from microarray technology, which presents challenges such as inconsistencies across different detection platforms and variability due to diverse specimen origins. These factors could potentially impact the reliability and generalizability of our findings. While our machine learning model has shown effectiveness in predicting SLE, further validation with external, independent datasets is imperative to solidify its predictive power. Lastly, our study is limited by the absence of experimental validation. Computational analyses, though powerful, are complemented by empirical experiments. Practical laboratory experiments and in-depth model evaluations are essential to corroborate the biological relevance and clinical applicability of our results.

In summary, this study provides the first evidence that the mitochondria-related genes FAM210B, MSRB2, LYRM7, IFI27, and SCO2 could be useful biomarkers for the classification and treatment of SLE. These findings contribute to our understanding of the role of mitochondrial dysfunction in SLE and highlight the need for further investigation in this area to improve classification and treatment, ultimately leading to improved patient outcomes.

### Electronic supplementary material

Below is the link to the electronic supplementary material.

Supplementary Data S1: Inventory of MRGs Derived from MitoCarta 3.0.

Supplementary Data S2: List of DEGs in SLE.

Supplementary Data S3: Interaction Data Between DEGs and MRGs Resulting in Identification of DE-MRGs in SLE.

Supplementary Data S4: TF Enrichment Analysis Results for Hub Genes.

Supplementary Data S5: miRNA-mRNA Interaction Data Based on Hub Genes.

Supplementary Material 6: Figure S1: Calibration and Decision Curve Analysis of the ANN Model for SLE Classification.

Supplementary Data S6: Drug Target Prediction Data for Hub Genes.

## Data Availability

The original contributions presented in the study are included in the article/supplementary material. We have uploaded all my source code and experimental data to a cloud disk (https://www.jianguoyun.com/p/DTnH9WsQ9djyCxjnmJYFIAA). Further inquiries can be directed to the corresponding author.
