# Supplementary figures and images for: Decoding the mitochondrial connection: development and validation of biomarkers for classifying and treating systemic lupus erythematosus through bioinformatics and machine learning
Source: BMC Rheumatol. 2023 Dec 4;7:44. doi: 10.1186/s41927-023-00369-0 (PMC10694981; doi:10.1186/s41927-023-00369-0)

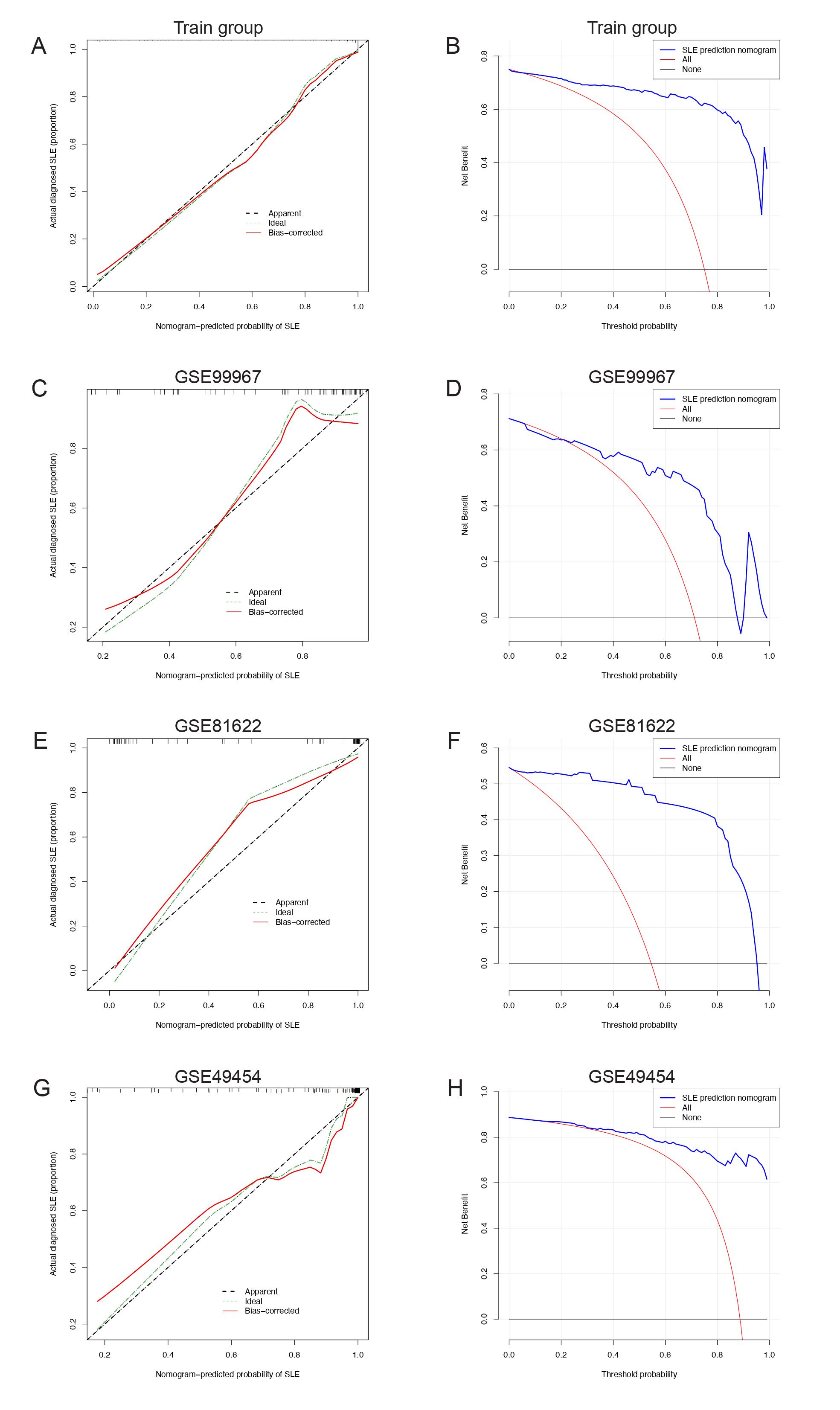

Supplement: Supplementary file 6 — Supplementary Material 6: Figure S1: Calibration and Decision Curve Analysis of the ANN Model for SLE Classification. [file 41927_2023_369_MOESM6_ESM.tif]
